# Uranium Isotope (U-232) Removal from Waters by Biochar Fibers: An Adsorption Study in the Sub-Picomolar Concentration Range

**DOI:** 10.3390/molecules27196765

**Published:** 2022-10-10

**Authors:** Maria Philippou, Ioannis Pashalidis, Charis R. Theocharis

**Affiliations:** Department of Chemistry, University of Cyprus, P.O. Box 20537, Nicosia 1678, Cyprus

**Keywords:** U-232, sub-picomolar range, modified biochar fibers, seawater, adsorption, *K*_d_ values

## Abstract

The adsorption of the U-232 radionuclide by biochar fibers in the sub-picomolar concentration range has been investigated in laboratory aqueous solutions and seawater samples. The adsorption efficiency (*K*_d_ values and % relative removal) of untreated and oxidized biochar samples towards U-232 has been investigated as a function of pH, adsorbent mass, ionic strength and temperature by means of batch-type experiments. According to the experimental data, the solution pH determines to a large degree the adsorption efficiency, and adsorbent mass and surface oxidation lead to significantly higher *K*_d_ values. The ionic strength and temperature effect indicate that the adsorption is based on the formation of inner-sphere complexes, and is an endothermic and entropy-driven process (ΔH° and ΔS° > 0), respectively. Regarding the sorption kinetics, the diffusion of U-232 from the solution to the biochar surface seems to be the rate-determining step. The application of biochar-based adsorbents to treat radioactively (U-232) contaminated waters reveals that these materials are very effective adsorbents, even in the sub-picomolar concentration range.

## 1. Introduction

The interaction of uranium with biochar-based adsorbents has been widely studied using different biomass types as starting materials [1,2,3,4], including plant fibers, which in addition to their abundance and low value present desirable adsorbent properties such as a tubular structure and large external surface, allowing for fast material exchange and increased sorption capacities [5,6,7,8,9]. These physical properties remain almost intact after carbonization and chemical oxidation by nitric acid. The latter, which is a chemical modification, results in adsorbent materials with enormous mechanical and chemical resistance, and specificity towards cationic species/metal ions due to the formation of surface carboxylic groups [6,7,10]. Moreover, the derivatization of surface moieties results in selectivity towards uranyl cations or other metal ions [11,12].

Uranium is a natural, ubiquitous element, the concentration of which varies depending on the geological background, and it is almost constant in oceans (~3.3 ppb) [13]. However, various activities including the nuclear fuel cycle and the use of depleted uranium have resulted in the production of huge amounts of uranium-containing solid and liquid waste, and in local environmental contamination by depleted uranium [14]. The uranium sorption by biochar fibers has been investigated with respect to uranium removal from contaminated waters using adsorption-based technologies, as well as for the recovery of uranium from industrial process and waste waters, to secure a long-term supply of uranium as fuel for nuclear power reactors [15].

The investigations related to uranium sorption by biochar-based materials have been performed in the micromole to millimole concentration range, which is relevant for studies that aim to determine the sorption capacity and carry out spectroscopic studies (e.g., Fourier-transform infrared (FTIR), Raman, X-ray photoelectron (XPS), etc.) for surface species characterization [6,7,10,11,12]. However, there are almost no studies in the nanomole to picomole range, which is for radionuclides of particular interest, because these can be hazardous to living organisms and human beings even at ultratrace levels. On the other hand, it is of particular interest to compare the sorption behavior in a wide concentration scale, including the effect of various parameters affecting the sorption efficiency (e.g., pH, temperature (T), ionic strength (I), adsorbent mass (m), etc.).

The present paper deals with the interaction of two different types of biochar fibers with uranium (e.g., U-232) in laboratory and seawater solutions at ultratrace levels (picomole range). The adsorption efficiency was expressed by the partition coefficient, *K*_d_, and investigated as a function of pH, adsorbent mass, ionic strength (I) and temperature.

## 2. Results and Discussion

### 2.1. The Effect of Contact Time on the U-232 Adsorption by LCC and LCC_ox

**Figure 1:**
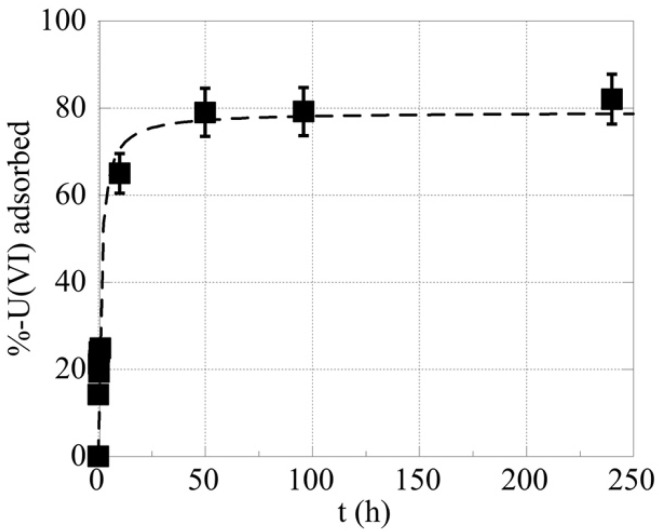
The %-relative amount of U(VI) adsorbed by oxidized biochar fibers (LCC_ox) as a function of time. [U-232] = 8.6 × 10^−14^ mol/L, biochar mass = 0.01 g, pH 4 and ambient conditions.

The effect of contact time was studied in order to evaluate the time needed to reach equilibrium in the system. The corresponding kinetic data are summarized in Figure 1 and indicate that equilibrium was reached after almost two days. Hence, the following experiments were performed after three days of contact time to assure equilibrium conditions. Compared to studies using higher uranium concentrations [6,8], in which equilibrium conditions were reached after a few hours of contact time, equilibrium in the sub-picomolar concentration range is achieved at significantly higher contact times (>50 h), because at such low concentrations, the diffusion of the uranium cations from the solution to the biochar surface is the adsorption rate-limiting step.

### 2.2. The Effect of pH on the U-232 Adsorption by LCC and LCC_ox

The solution pH was expected to affect to sorption efficiency (*K*_d_ values) because the proton concentration determines the U(VI) speciation in solution and the surface charge of the biochar materials. The latter is associated with π-proton interaction [16] and carboxylic dissociation [2] for the LCC and LCC_ox, respectively. Regarding U(VI), the predominant species in solution at pH 2 and pH 4, pH 7, and pH 9 were expected to be UO_2_^2+^, UO_2_CO_3_, and UO_2_(CO_3_)_3_^4−^, respectively [15]. On the other hand, at pH 2, the biochar surface carboxylic moieties are extensively protonated (pKa < 4) and partly deprotonated at pH 4. Only at pH 7 and pH 9 are the carboxylic groups extensively deprotonated and the surface negatively charged [6].

**Figure 2:**
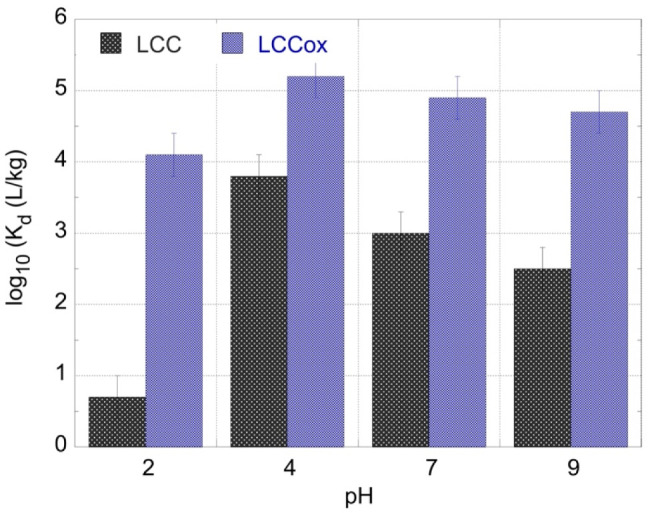
The *K*_d_ values for the U(VI) adsorption by two different types of biochar fibers as a function of pH. [U-232] = 8.6 × 10^−14^ mol/L, biochar mass = 0.01 g and ambient conditions.

According to Figure 2, the lowest sorption efficiency (log_10_*K*_d_ (LCC) = 0.7 ± 0.3 and log_10_*K*_d_ (LCC_ox) = 4.1 ± 0.3) is observed at pH 2, which is related to the competitive interaction of protons with the π-system of LCC and the partial deprotonation of the carboxylic moieties on the LCC_ox surface, respectively. The highest *K*_d_ values are observed at pH 4 (log_10_*K*_d_(LCC) = 3.8 ± 0.3 and log_10_K (LCC_ox) = 5.2 ± 0.3) because of the partial deprotonation of the surface moieties, which attract the uranyl cation (UO_2_^2+^) to form the π–uranyl cation and U(VI)-carboxylate surface species. Furthermore, at pH 7 (log_10_*K*_d_(LCC) = 3.0 ± 0.3 and log_10_K (LCC_ox) = 4.9 ± 0.3) and pH 9 (log_10_*K*_d_(LCC) = 2.5 ± 0.3 and log_10_K (LCC_ox) = 4.7 ± 0.3) the adsorption efficiency declines gradually due to the stabilization of U(VI) in solution in the form of the stable U(VI)-carbonato species (UO_2_CO_3_ and UO_2_(CO_3_)_3_^4−^), and the repulsive forces between the negatively charged U(VI) species and the biochar surface, particularly in the case of LCC_ox.

By a comparison of the pH effect observed in this study with previous studies performed at significantly higher concentration (six to nine orders of magnitude) [6,7,8,9], it is obvious that the effect of pH is similar, assuming that the sorption chemistry is governed in both cases by the surface characteristics of the adsorbent and the U(VI) speciation in solution.

### 2.3. The Effect of Adsorbent Mass on the U-232 Adsorption by LCC and LCC_ox

In order to evaluate the effect of the adsorbent mass on the sorption efficiency, adsorption experiments were performed using similar solutions ([U-232] = 8.6 × 10^−14^ mol/L, pH 2) and varying adsorbent masses. These experiments were performed at a pH of 2 in order to obtain, in all cases, a relative removal below 100% for a better comparison.

**Figure 3:**
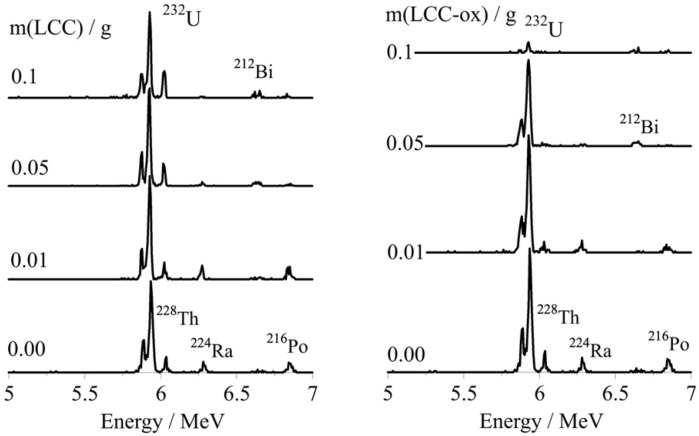
The alpha spectra of the U-232-traced aqueous solutions after treatment with different masses of unmodified (m(LCC) in g) and oxidized biochar (m(LCC_ox) in g). [U-232] = 8.6 × 10^−14^ mol/L, pH 4 and ambient conditions. It has to be noted that Bi-212, Ra-224, Th-228 and Po-216 are daughter nuclides of U-232 and are therefore present in the alpha spectra.

The activity concentration of the remaining U-232 in solution was determined by alpha-spectroscopy and the corresponding spectra are summarized in Figure 3, indicating the effect of the biochar mass on the sorption efficiency, particularly in the case of LCC_ox.

**Figure 4:**
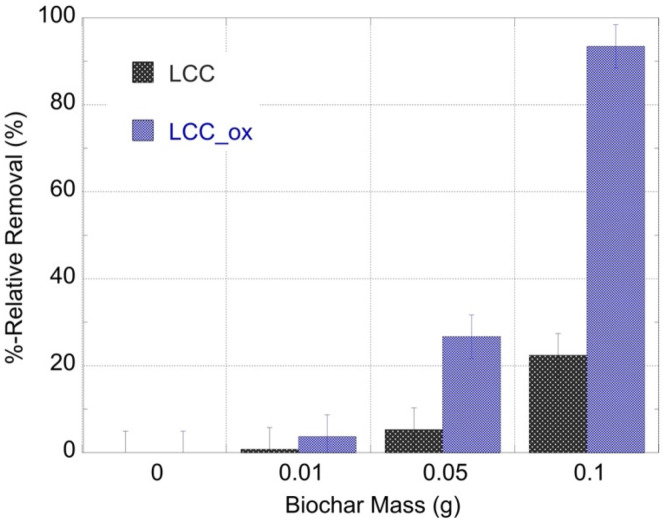
The %-relative removal efficiency of U-232-traced aqueous solutions after treatment with different masses of unmodified (LCC) and oxidized biochar (LCC_ox). [U-232] = 8.6 × 10^−14^ mol/L, pH 4 and ambient conditions.

The alpha-spectroscopic data were evaluated to calculate the relative U(VI) removal and the corresponding data are graphically presented in Figure 4. It is obvious that the sorption efficiency increased exponentially with the adsorption mass and the highest relative removal values are observed for LCC_ox. This is in agreement with previous studies performed at elevated concentrations, indicating the affinity of biochar materials for U(VI) and the importance of surface modification (e.g., surface oxidation) to improving the affinity and adsorption capacity of the biochar materials towards metal/metalloid species (e.g., uranium) in aqueous solutions [6,7,8,9].

### 2.4. The Effect of Ionic Strength on the U-232 Adsorption by LCC and LCC_ox

At increased metal ion concentrations, spectroscopic methods (such as FTIR, Raman and XPS) may be used to evaluate the adsorption mechanism at the molecular level [5,6,7,8,9,12,16,17,18]. However, this was impossible at the ultratrace levels used in the present study and therefore the effect of the ionic strength on the adsorption efficiency was employed to indicate the type of surface complexes formed (e.g., inner- or outer-sphere complexes). Generally, the decline of the adsorption efficiency with increasing ionic strength indicates non-specific/electrostatic interactions and the formation of outer-sphere complexes. On the other hand, insignificant changes in the adsorption efficiency with increasing ionic strength are associated with the formation of specific interactions and the formation of inner-sphere complexes [6,7].

**Figure 5:**
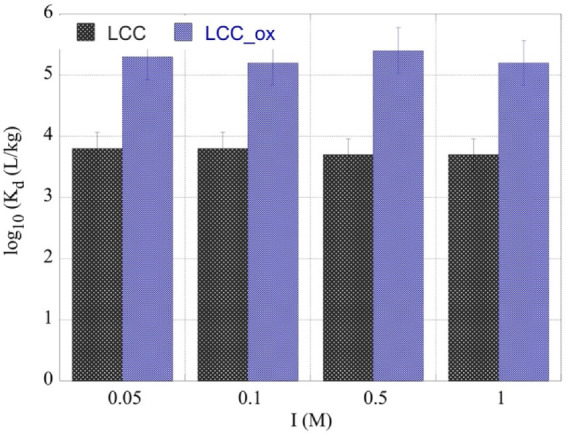
The *K*_d_ values for the U(VI) sorption by two different types of biochar fibers (LCC and LCC_ox) as a function of ionic strength. [U-232] = 8.6 × 10^−14^ mol/L, biochar mass = 0.01 g, pH 4 and ambient conditions.

According to the data in Figure 5, which show the adsorption efficiency (log_10_*K*_d_ values) as a function of the ionic strength, there was almost no effect of the ionic strength on adsorption efficiency, assuming specific interactions and formation of inner-sphere complexes between the uranyl cation and biochar surface moieties, which are carboxylic/carboxylate groups particularly on the surface of LCC_ox. It has to be noted that oxygen-containing moieties were also present (to a lesser extent) on the surface of untreated biochar contributing significantly to U(VI) adsorption by LCC, particularly at pH 4.

### 2.5. The Effect of Temperature on the U-232 Adsorption by LCC and LCC_ox

The evaluation of the thermodynamic parameters (ΔH^0^; and ΔS^0^) for the U(VI) adsorption by the two different biochar materials was performed by determining the corresponding *K*_d_ values at three different temperatures and plotting ln*K*_d_ versus 1/T according to the Van ’t Hoff equation:

(1) $${\ln K}_{d}=-\frac{\Delta H^{0}}{R\cdot T}+\frac{\Delta S^{0}}{R}$$

**Figure 6:**
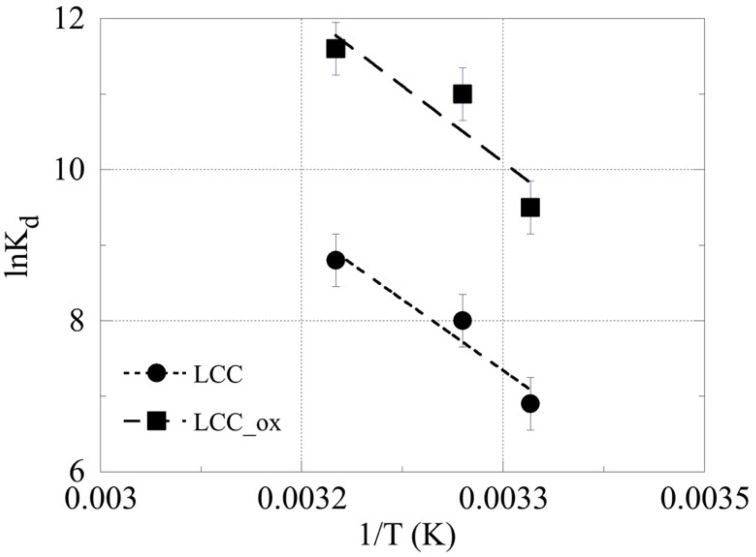
The ln*K*_d_ as a function of 1/T for the sorption of U(VI) by LCC and LCC_ox, at an initial uranium concentration of 8.6 × 10^−14^ mol/L, biochar mass = 0.01 g, pH 4 and 3 days of contact time.

The ln*K*_d_ −1/T plot is shown in Figure 6; the thermodynamic parameters were calculated using the slope and intercept obtained from the linear regression of the associated experiment, and were found to amount to ΔH^0^ = 1.4 kJ mol^−1^ and ΔS^0^ = 5.4 J K^−1^ mol^−1^, and ΔH^0^ = 1.5 kJ mol^−1^ and ΔS^0^ = 6.1 J K^−1^ mol^−1^, for the adsorption of U(VI) by LCC and LCC_ox, respectively. These data clearly indicate that the adsorption of U(VI) by both biochar materials is an endothermic and entropy-driven process, and are in agreement with previous studies. However, the values obtained are up to two orders lower than the corresponding values obtained from experiments performed using higher uranium concentrations [6,7]. This is attributed to the fact that at higher concentrations, the assumption of a large excess of binding sites compared to the metal species is not viable, resulting in those significant differences.

### 2.6. The Removal of U-232 from Seawater by LCC and LCC_ox

**Figure 7:**
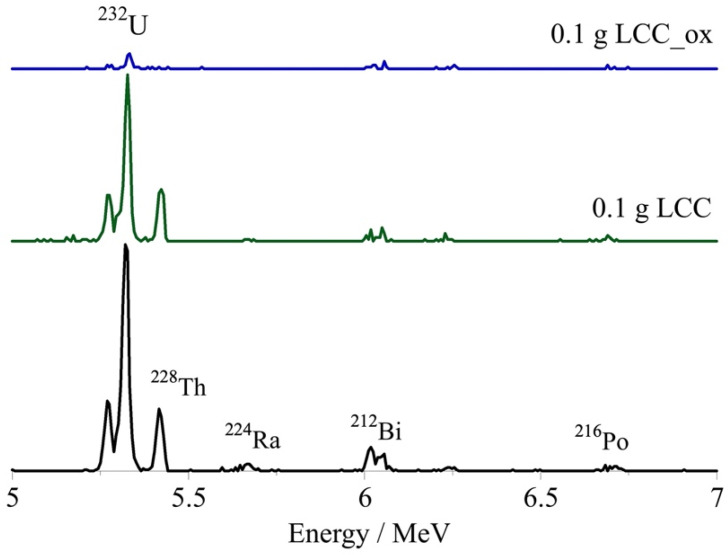
The alpha spectra of U-232-traced seawater solutions treated with 0.1 g of unmodified (LCC) and oxidized biochar (LCC_ox). [U-232] = 8.6 × 10^−14^ mol/L, pH 8.3 and ambient conditions.

**Figure 8:**
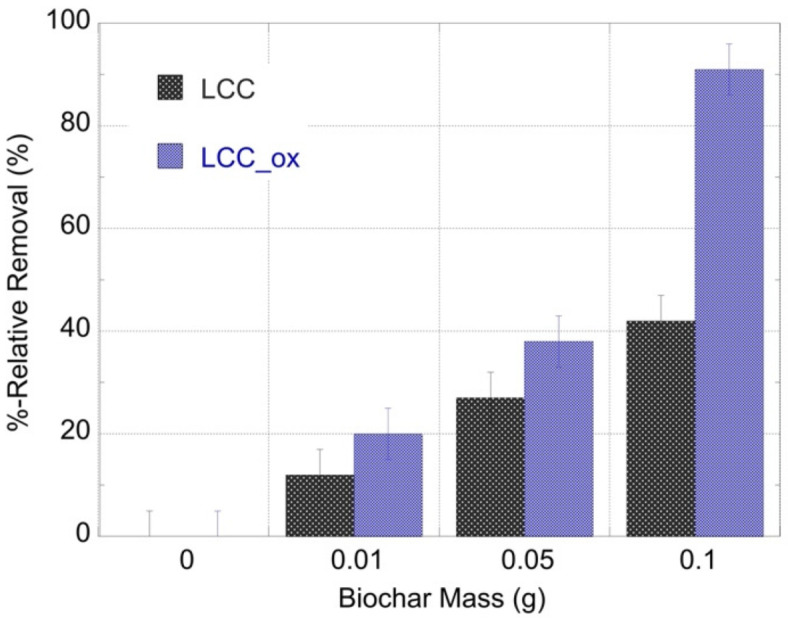
The %-relative removal efficiency of U-232-traced seawater solutions after treatment with different masses of unmodified (LCC) and oxidized biochar (LCC_ox). [U-232] = 8.6 × 10^−14^ mol/L, pH 8.3 and ambient conditions.

The applicability of the biochar materials regarding the removal of ultratrace amounts of the radionuclide from natural waters was tested using U-232-contaminated seawater solutions and varying biochar amounts (e.g., 0.01, 0.05 and 0.1 g). After 24 h of contact, the remaining radionuclide concentration in solution was determined by alpha-spectroscopy and the representative spectra are shown in Figure 7. The relative U-232 removal from the seawater solution (20 mL) using various amounts of biochar is graphically summarized in Figure 8. The data (Figure 7) clearly show that increasing biochar mass resulted in increasing removal efficiency and that the oxidized counterpart (LCC-ox) presented a higher removal efficiency, which was almost 100% for the seawater sample treated with 0.1 g.

Compared to the corresponding data obtained from laboratory solutions (pH 2), it is obvious that the removal efficiency in seawater solutions is generally higher for both biochar materials (LCC and LCC_ox) despite the presence of competing cations (e.g., Ca^2+^, Fe^3+^) and complexing ligands (e.g., CO_3_^2−^) that disfavor U(VI) surface complexation [6,7]. This is ascribed to the higher pH of seawater solutions compared to the laboratory solutions (pH 2), and the associated higher affinity of the surface moieties for U(VI) at pH 8.3 compared to pH 2. Nevertheless, at lower adsorbent amounts, the removal efficiency decreases dramatically, because the surface active sites are quantitatively occupied by competing cations (e.g., Ca^2+^, Fe^3+^), indicating that the materials are non-selective adsorbents and can bind polyvalent metal ions that can specifically interact and form complexes with the carboxylic moieties, present particularly on the LCC_ox surface.

## 3. Materials and Methods

All experiments were carried out in 30-mL polyethylene (PE) screw-capped bottles under ambient conditions (23 ± 2 °C). The uranium isotope, U-232 (t_1/2_ = 68.9 years), was used for the studies. Reference and test solutions had the same initial activity concentration (0.5 mBq/mL) and were prepared from a standard tracer solution (4.923 kBq/g) purchased from NPL (United Kingdom). The studied biochar samples included biochar prepared from *Luffa cylindrica* fibers (LCC) and its surface-oxidized counterpart (LCC_ox). The preparation and characterization of unmodified and oxidized biochar fibers is extensively investigated and described elsewhere [6,7]. In contrast to LCC, which is characterized by a graphite-like surface, the carboxylic moieties govern the surface charge and chemistry of LCC_ox. The experiments were performed in laboratory solutions using deionized water of different pH (i.e., 2, 4, 7 and 9) and in seawater collected from a local coastal area. The composition of the seawater samples is given elsewhere [19]. The pH was adjusted using HCl and NaOH solutions, and measured using a combined glass electrode and a pH meter (Hanna Instruments, Woonsocket, RI, USA), which was calibrated prior to each experiment with buffer solutions (Scharlau, Barcelona, Spain). The radiometric analysis of the uranium isotope (U-232) was performed by means of an alpha-spectrometer (Canberra France, Loches, France), as described elsewhere [20]. In addition, the reference and control samples from the U-232 analysis were obtained by liquid scintillation counting (LSC, Triathler, Hidex, Turku, Finland). Alpha-spectrometry measurements were carried out in duplicate and LSC measurements were also performed in parallel to compare the data obtained from both radiometric methods. The detection limits were evaluated to be 0.05 mBq and 0.03 mBq for the LSC and alpha-spectrometric measurements, respectively.

The adsorption studies were carried out by contacting 0.01 g of the biochar with 20 mL of the U-232 solution at an activity concentration of 25 Bq/L ([U-232]= 8.6 × 10^−14^ mol/L) in 30-mL screw-capped PE bottles. Specifically, the effect of contact time was explored at a pH of 4 and under the previously mentioned conditions. Similarly, the pH effect was investigated for the pH of 2, 4, 7 and 9. The effect of the biochar mass was investigated in laboratory solutions (pH 2) and seawater solutions (pH 8.3) by adding 0.01, 0.05 and 0.1 g of biochar and the reference solution (without biochar). The effect of ionic strength (I) was considered using aqueous NaClO_4_ solutions of varying concentrations (0.05, 0.1, 0.5 and 1Μ). The effect of temperature was investigated at 25, 40 and 60 °C at a pH of 4. The suspensions were agitated on a rocking shaker (SK-R1807, DLAB, Beijing China) at an agitation rate of 65 min^−1^. For the uranium analysis, aliquots of 200 μL were used to determine the radionuclide concentration in solution using an alpha-spectrometer or a liquid scintillation counter, which were previously calibrated using standard reference solutions and sources.

The partition coefficient, *K*_d_, was used to evaluate the sorption efficiency, because of the sub-picomolar uranium concentrations used and the great excess of surface binding sites (B). The partition coefficient, *K*_d_, is defined as:

*K*_d_ = [U(VI)]_ads_/[U(VI)]_aq_ (L/kg) (2)

where [U(VI)]_ads_ (Bq/g) is the activity of the U-232 adsorbed by the biochar and [U(VI)]_aq_ (Bq/L) is the U-232 activity concentration in solution at equilibrium. The amount of U-232 adsorbed by biochar (dry mass) was calculated from the total activity of U-232 adsorbed minus the activity of U-232 adsorbed by the plastic bottle walls, which was not negligible and had to be taken into account. In addition, under ambient conditions, the U(VI) was expected to be the predominant oxidation state of uranium in solution [21].

In addition, the sorption efficiency is expressed as %-relative removal and is calculated by:

%-relative removal = 100·([U(VI)]_R_ − [U(VI)]_aq_)/[U(VI)]_R_) (3)

where [U(VI)]_R_ is the U-232 concentration in the reference solution.

The experiments were performed in triplicate, and the mean values and uncertainties were used for the data evaluation and graphical presentations.

## 4. Conclusions

Biochar materials have the ability to adsorb radionuclides (U-232) even in the sub-picomolar concentration range. The adsorption efficiency strongly depends on the solution pH that governs surface charge and U(VI) speciation in solution. Surface modification, such as the oxidation of the biochar, results in a significantly higher sorption capacity and affinity of the adsorbent for U(VI) due to the formation of surface carboxylic moieties. Variation of the ionic strength does not have any significant effect on the sorption efficiency, indicating the formation of inner-sphere surface complexes, and the evaluation of the data obtained from the experiments performed at different temperatures revealed endothermic and entropy-driven adsorption processes. In seawater, that has a pH~8.3 and contains anions (e.g., Ca^2+^, Fe^3+^) which can compete with U(VI) and occupy sorption sites on the MPs surface, and anions (CO_3_^2−^, SO_4_^2−^) which can act as ligands and stabilize U(VI) in the aqueous phase, the sorption efficiency decreases dramatically. Nevertheless, increasing the biomass amount, particularly in the case of LCC_ox, can result in the almost quantitative removal of the radionuclide even from seawater solutions. 

Future studies could include experiments related to the adsorption efficiency of biochars for other radionuclides (e.g., Ra, Am, Pu) and surface derivatization to enhance selectivity towards certain radionuclides of particular interest, especially for use in nuclear medicine, as well as experiments to study the competitive adsorption in radionuclide mixtures.

## Data Availability

Not applicable.
